# Validity and reliability of a questionnaire assessing Brazilian adolescents’ experiences in orthodontic treatment

**DOI:** 10.1590/1807-3107bor-2026.vol40.033

**Published:** 2026-06-12

**Authors:** Tatiana Ettore do Valle de Sousa FREITAS, Marina Bernardes Grillo de BRITO, Mariana Caires Sobral de AGUIAR, José Augusto Mendes MIGUEL

**Affiliations:** (a) Marinha do Brasil, Navy Central Dental Clinic, Rio de Janeiro, RJ, Brazil.; (b) Private Orthodontics Office, Luxembourg, Luxembourg.; (c) Universidade do Estado do Rio de Janeiro – UERJ, School of Dentistry, Department of Preventive and Community Dentistry, Rio de Janeiro, RJ, Brazil.

**Keywords:** Orthodontics, Validation Study, Surveys and Questionnaires

## Abstract

The study aimed to validate a questionnaire for the Brazilian adolescent population assessing patient’s perceptions of orthodontic treatment and to verify whether its original psychometric properties were preserved during the transcultural adaptation process. A total of 160 adolescents, assessed before and after orthodontic treatment at two different orthodontic clinics,answered a self-administered questionnaire translated into Portuguese. Patients were equally assigned to two groups: non-treated adolescents (NTA) and adolescents receiving orthodontic treatment (AOT). Both male and female adolescents were selected (age range: 12 to 17 years). A trained orthodontist examined all patients to record the aesthetic dental index (ADI) and the decayed, missing, and filled teeth (DMFT) index. The adolescents’ legal guardians answered a socioeconomic questionnaire. Reliability was assessed by evaluating the instrument’s internal consistency and stability, using Cronbach’s alpha, the intraclass correlation coefficient (ICC), and Cohen’s kappa coefficient (k). Construct validity was assessed through cross-cultural validation and hypothesis testing. Cronbach’s alpha was 0.7 for the NTA group and 0.9 for the AOT group. The instrument demonstrated excellent stability for the NTA group, yielding an ICC > 0.9. The k values showed a strong to almost perfect correlation (between 0.701 and 0.999) for the AOT group. The Brazilian version of the QPAOT proved to be a reliable and stable instrument, with good psychometric properties and can therefore be considered an appropriate instrument for assessing adolescents’ perceptions and expectations regarding orthodontic treatment.

## Introduction

The success of orthodontic treatment relies not only on the orthodontist’s expertise but also on the interaction of several factors that could limit the course of treatment. One of these factors is the inherent complexity of each case and, in particular, the level of patient cooperation. Thus, to achieve successful treatment, efficient techniques that require minimal patient cooperation, and minimize pain and discomfort.

A growing number of questionnaires or tools are now available to assess psychosocial factors and various health outcomes in research, clinical practice, and population health studies. Nonetheless, only few instruments evaluate patients’ expectations regarding orthodontic treatment, which can directly influence their treatment satisfaction and adherence.^1,2^

Feldman et al.^3^developed and validated the Questionnaire about Perceptions of Adolescents in relation to Orthodontic Treatment (QPAOT), designed to assess adolescent patients’ perceptions tjhroughout the course of orthodontic treatment , from the initial decision to start treatment to satisfaction with the outcomes. Since its development, QPAOT has been used in clinical trials evaluating various orthodontic therapies to assess patients’ perceptions throughout orthodontic treatment.^4-7^ The original questionnaire was validated in English only and has been translated and culturally adapted to be used in orthodontic research in Portuguese-speaking countries.^8^

Ideally, orthodontic research on the efficacy of new therapies should include normative data and assessment of patients’ perceptions. To apply this instrument for assessing expectations and perceptions of orthodontic patients in Braziland to allow for comparisons with previous research, it is necessary to validate the previously translated version of the questionnaire.^8^ Therefore, the aim of the present study was to validate the Portuguese version of the QPAOT developed by Feldman and to test its reliability and reproducibility by assessing internal consistency and stability. The aim was also to evaluate adolescents’ perception prior to and during orthodontic treatment in association with age, sex, dental aesthetic index (DAI), decayed, missing, and filled teeth (DMFT) index, and socioeconomic background.

## Methods

### Cross-cultural adaptation and pilot study methods

The authors obtained permission via email from the original developer of the questionnaire.^3^The translation and cross-cultural adaptation processes have been previously described.^8^

The methodology used assumed the universalist perspective of cross-cultural equivalence of measurement instruments, as proposed by Herdman et al.^9^ Before translating the questionnaire, the conceptual model and item relevance were assessed in the Brazilian cultural context by a panel of experts (two experienced orthodontists, a graduate student in Orthodontics, and a dentist experienced in developing and adapting quality-of-life instruments). Following the established guidelines, the adaptation included six steps: independent forward translation, pretesting, consolidation, back-translation, review, and synthesis.^10-12^

The questionnaire was initially translated into Portuguese by two independent translators, both native Portuguese speakers fluent in English, with no prior knowledge of the instrument and no communication between them during the process. The expert panel then met to produce a single, consensus-based version, selecting the most appropriate wording and culturally adapting the content for Brazilian adolescents. This step involved evaluating semantic and cultural equivalence, as well as operational equivalence (i.e., adapting procedures to ensure usability was comparable to that of the original instrument).

The synthesized Portuguese version was then back-translated into English by two independent bilingual translators unaware of the instrument’s objectives, to avoid bias. They discussed discrepancies and consolidated a final back-translation. Based on these results, the expert panel proposed a final Portuguese version, which was used in a pilot study to assess additional types of equivalence.^8^

The study was approved by the local Research Ethics Committee. All patients and their legal guardians were informed about the characteristics and objectives of the research and signed the assent form and the informed consent form. Prior to the beginning of the study, the authors of the original questionnaire were consulted and authorized the project. This was a cross-sectional study with non-probabilistic and convenience sampling. Participants were aged 12 to 17 years and were native Portuguese speakers. Patients with labiopalatal fissure, craniofacial syndromes or congenital diseases with dental anomalies, agenesis, or supernumerary teeth were excluded. Patients were equally assigned to two groups: non-treated adolescents (NTA) and adolescents receiving orthodontic treatment (AOT).

### Questionnaires and indices

The following instruments were used in the present study:

- P*ortuguese version of the QPAOT*: a 46-item questionnaire organized into five domains: (1) motivation for treatment – 7 items; (2) expectations about treatment (4 items); (3) pain and discomfort in teeth, maxillary jaws and face – 13 items; 4) maxillary functional impairment – 18 items; (5) and questionnaire validity – 4 items. In the two first domains, responses were available on a numerical scale from 0 to 10, with the extremes ranging from “nothing” to “much” or “nothing” to “completely.” In the pain domain, the first 10 questions used the same scale, but with extremes ranging from “none” to “the worst possible.” Question 11 was answered using a dichotomous scale (yes or no) and questions 12 and 13 were assessed on a three-point scale. The fourth domain evaluates functional impairment using a four-point Likert scale, with response options defined as “nothing,” “slightly,” “a lot,” or “extremely.” Instrument validity was also assessed on a numerical scale from 0 to 10, with the extremes defined as “nothing” and “a lot.” Domains 1 and 2 were evaluated in the NTA group, whereas domains 3 and 4 were applied to the AOT group. Questions 1 and 2 from the validation domain were analyzed in the NTA group and questions 3 and 4 in the AOT group (Figures 1, 2, and 3).
  **Figure 1:**
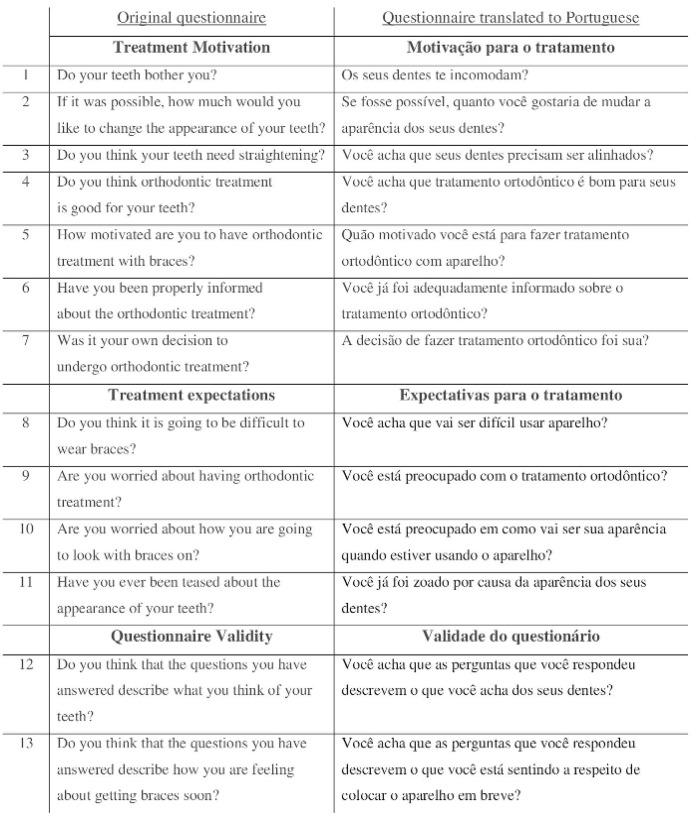
Questionnaire applied to the non-treated adolescents’ group.
  **Figure 2:**
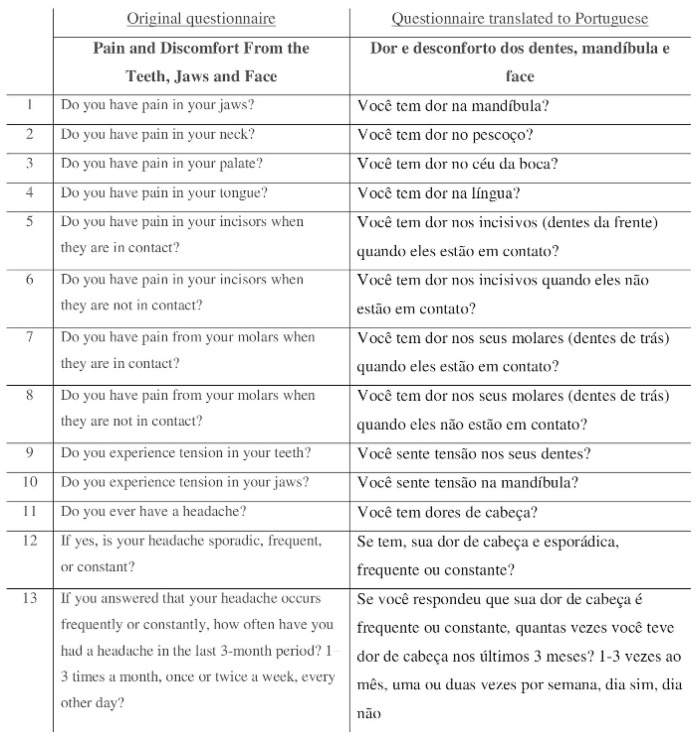
Questionnaire applied to the treated adolescents’ group – First part.
  **Figure 3:**
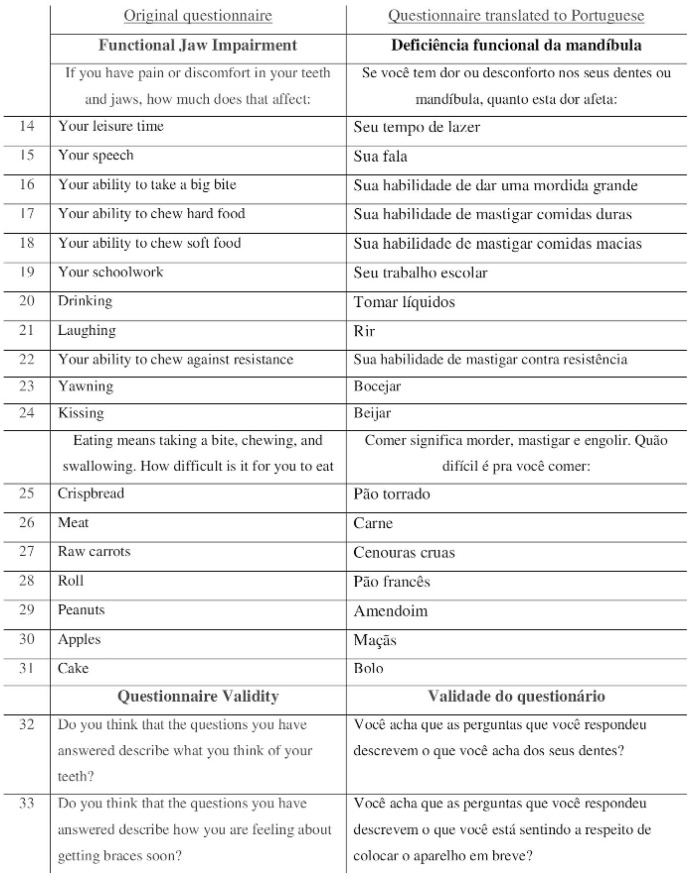
Questionnaire applied to the treated adolescents’ group – Second part.
- *DAI*: This quantitative indicator of orthodontic treatment need assessed the degree of aesthetic impact of the dentition. This index evaluated 10 occlusal characteristics that are socially relevant for dental aesthetics: tooth absence, anterior crowding, anterior spacing, midline diastema, most anterior irregularity in the maxilla, most anterior irregularity in the mandible, maxillary overjet, negative overjet, anterior open bite, and anteroposterior molar relationship. The measurements were performed by a single previously calibrated operator within three minutes.
- *DMFT:* This is a quantitative index of caries experience in a given population. In the present study, this index was applied individually to each patient. The DMFT index was evaluated using a mouth mirror and artificial lighting with patients seated in a dental chair at the Orthodontic Clinic of UERJ and at the Central Navy Dental Clinic. Data collection included teeth lost to caries, teeth with carious lesions, and restored teeth. All examinations were performed by a previously calibrated operator and were completed in approximately two minutes.
- *Patients’ socioeconomic classification*: Obtained from the Brazilian Economic Classification Criterion of the Brazilian Association of Research Companies and used to estimate the purchasing power of individuals and families in the urban area. The classification categorizes the market exclusively into economic classes (A, B1, B2, C1, C2, D, and E). The present study used only classes A, B, C, and D for statistical purposes.

The pilot study included 20 consecutive patients, selected according to the previously described inclusion and exclusion criteria.^8^ Two calibrated researchers conducted the pilot study, carrying out the steps of the instrument validation test to verify the feasibility of its planned implementation. Both researchers were present during the administration of the pilot questionnaire to all 20 patients, ensuring standardization of the method and its presentation to the study participants.

To test psychometric properties (validity and reliability) of the QPAOT, the questionnaire was applied to 160 adolescents, equally divided into two groups (NTA and AOT). Sample size calculation followed the ratio of interviewed individuals per item, with participants selected from two different Orthodontic clinics.

After explaining the aims of the research and obtaining consent from patients and their legal guardians, a single previously calibrated operator performed the clinical examinations to record the DAI index and the DMFT index. The operatoralso explained how to complete each questionnaire. Socioeconomic classification data were provided by the legal guardians.

### Statistical analysis

Statistical analyses were conducted using IBM SPSS Statistics 21^®^ (IBM Corp., Armonk, USA). The Shapiro-Wilk test was applied to assess the normality of the data. Descriptive statistics were performed for each item, including age, age group, sex, institution where the participant was interviewed, DAI score, socioeconomic status, and educational level of the legal guardian. Means, standard deviations (SD), and minimum and maximum values were calculated for continuous variables. Frequencies were calculated for categorical variables, corresponding to each impact included in the QPAOT.

Instrument stability was assessed through test–retest reliability, applying the intraclass correlation coefficient (ICC) for continuous variables and Cohen’s kappa (κ) for categorical variables. Internal consistency was evaluated using Cronbach’s alpha for each domain, both individually and for the questionnaire as a whole (including alpha values for each excluded item). Corrected item-total correlations were also calculated.

Associations between QPAOT scores and sex, age group, and DMFT index were assessed using t-tests, while one-way ANOVA was employed to assess differences according to DAI scores and socioeconomic classification.

Instrument validity was evaluated in terms of construct and face validity. Construct validity was tested using a hypothesis-driven analysis based on participant age and malocclusion severity. Two hypotheses were formulated: a) older participants would report higher QPAOT scores than younger ones; and b) participants with greater treatment needs (as indicated by DAI scores) would also report higher QPAOT scores. Table 1 presents the criteria used to assess these hypotheses. Face validity was determined using the method proposed in the original instrument, in which participants evaluated the relevance of each questionnaire item after responding.

**Table 1:** Criteria for testing construct validity using hypothesis testing.

Hypothesis	Variable	Measurement
1) Patients with higher DAI scores will show higher scores across all domains.	DAI	1 - No necessity
A more severe malocclusion causes greater aesthetic and functional impairment		2 - Elective
		3 - Highly desirable
		4 - Essential
2) Older patients (aged between 15 and 17 years) will have greater impact on their treatment expectations and motivations (domains 1 and 2)	Age range	1 – From 12 to 14 years
(Older patients have a greater perception of their aesthetics and dental impairment)		2 - From 15 to 17 years

## Results

### Study population characteristics

The sample included 160 adolescent patients, with 80 in the pretreatment group and 80 in the active orthodontic treatment group. The mean age was 14.4 years (SD: 1.73) for both groups. Eighty-seven patients (54%) were female and 73 (46%) were male.

According to the DAI index, patients requiring essential treatment were the most frequent (n = 52. 33%), and most of them belonged to class C (n = 86. 54%) and B (n = 63. 39%). The education level of the household head was predominantly “high school/ incomplete undergraduate” (n =112, 70%) and “completed undergraduate” (n = 29, 18%) (Table 2).

**Table 2:** Sociodemographic characteristics of the sample

Variable	Total		NTA		AOT	
	n	%	n	%	n	%
Total	160	100	80	50	80	50
Sex						
Male	73	46	37	46	36	45
Female	87	54	43	54	44	55
Institution						
OCM	66	41	26	33	40	50
UERJ	94	59	54	68	40	50
Age range						
1	82	51	41	51	41	51
2	78	49	39	49	39	49
DAI						
No Necessity	42	26	17	21	25	31
Elective	44	27	24	30	20	25
Highly desirable	22	14	11	14	11	14
Essential	52	33	28	35	24	30
Socioeconomic class						
A	0	0	0	0	0	0
B	63	39	30	37	33	41
C	86	54	43	54	43	54
D	11	7	7	9	4	5
Legal guardian						
Illiterate	5	3	5	6	0	0
Elementary II Incomplete	6	4	6	7	0	0
Completed High School	112	70	47	59	65	81
Incomplete high school	8	5	3	4	5	6
Completed undergraduate	29	18	19	24	10	13

The clinical examination showed that patients in the active treatment group had a mean DMFT index of 0.66, on a scale ranging from 0 (lowest) to 5 (highest). Patients in the pretreatment group had a mean index of 0.96, on a scale from 0 (lowest) to 7 (highest). The questionnaire scores for the NTA group ranged from 48 to 109, with a mean of 77 (SD = 13.04). In the AOT group, the scores ranged from 17 to 133, with a mean of 39.8 (SD = 24.2).

In the domain assessing orthodontic treatment expectations, the item with the greatest impact was “Are you concerned about how you will look when you are wearing orthodontic appliances?”, with a mean score of 5.81. Table 3 shows the mean impacts of each item in the domain, with the respective standard deviations and minimum and maximum values obtained in the sample.

**Table 3:** Median values assessed in the face validity domain (NTA Group). n = 80.

Face Validity Domain	
Question	Média
Do you think that the questions you have answered describe what you think of your teeth?	8.65
Do you think that the questions you have answered describe how you are feeling about getting braces soon?	8.54

The functional impairment of the jaws domain was assessed through the frequency of the impacts of its items, given that the items were rated on a Likert scale. The items with the greatest functional impact were “their ability to chew hard foods” and “their ability to take a big bite,” with nine and five patients, respectively, reporting the highest scores. Regarding the impact on diet, the items with the highest scores were apples, raw carrots, and meat, with six, six, and five patients, respectively, achieving the highest scores (Table 4).

**Table 4:** Mean values assessed in the face validity domain (AOT group). n = 80.

.	
Do you think that the questions you have answered describe what you think of your teeth?	9.15
Do you think that the questions you have answered describe how you are feeling about getting braces soon?	9.24

### Questionnaire validity

The mean scores of the questions used to assess face validity are shown in Tables 3 and 4. The high scores indicate that the participants considered the questions to be relevant.

Discriminant validity was evaluated by testing previously established hypotheses regarding the questionnaire’s ability to discriminate groups with different levels of motivation and expectations for treatment, pain and discomfort, functional impairment of the jaws, related to the severity of malocclusion (assessed by the DAI index) and age group. The associations were tested using ANOVA for the DAI index and the t-test for age groups.

**Figure 4:**
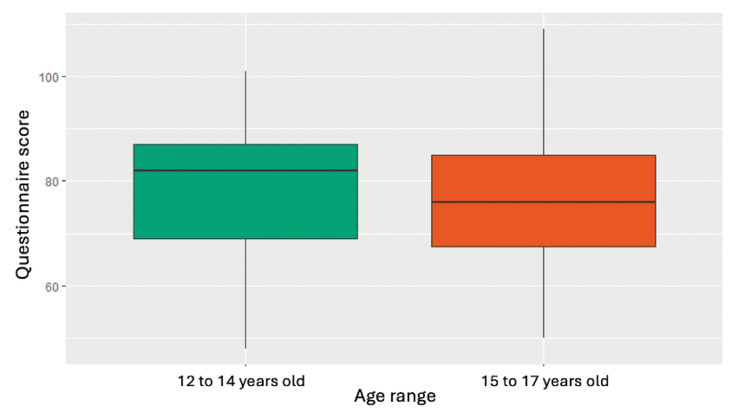
Percentile distribution of QPAOT scores in the NTA group according to age group.

**Figure 5:**
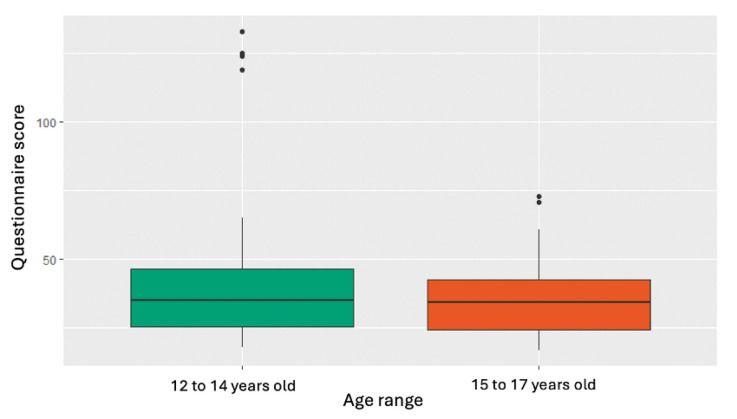
Percentile distribution of QPAOT scores in the AOT group according to age group.

In the NTA group, a strong association was found between the sum of the scores in the motivation and treatment expectations domains and the DAI index: the greater the need for orthodontic treatment, the greater the motivation and expectation to undergo treatment. In the AOT group, an association was also found between the sum of the domain scores in the pain and functional impairment of the jaw domains and the DAI index: the greater the need for orthodontic treatment, the greater the sensation of pain and functional impairment of the jaw reported by the patient. Data are shown in Figures 4 and 5, respectively.

**Figure 6:**
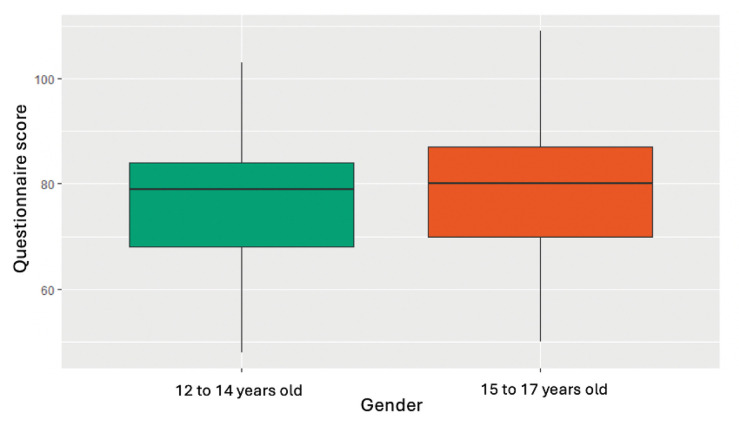
Percentile distribution of QPATO scores in the NTA group according to sex.

**Figure 7:**
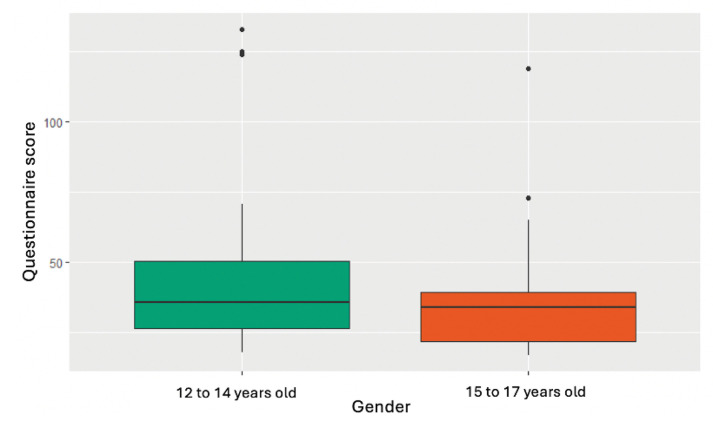
Percentile distribution of QPATO scores in the AOT group according to sex.

Neither the NTA nor the AOT group showed an association between the sum of scores in the motivation and treatment expectations domains and the patient age group, at a significance level of 5% (Figures 6 and 7).

### Internal consistency

The analysis of reliability by Cronbach’s alpha demonstrated that the internal consistency of the questions administered to the patients before treatment was satisfactory, while the questions applied to patients in the active treatment group showed excellent consistency (Cronbach’s α = 0.7 and 0.92 respectively), as shown in Table 5.

**Table 5:** Internal consistency of Cronbach’s α for each domain of the QPAOT.

Domains	Cronbach’s α	Lower Confidence Limit (95%)
Treatment motivation	0.711	0.62
Treatment expectations	0.698	0.67
Pain and discomfort of teeth, mandible, and face	0.917	0.89
Jaw functional impairment	0.91	0.88
Questionnaire validity	0.89	0.78

### Questionnaire stability

Instrument stability was measured through test-retest reliability, utilizing ICC and the kappa coefficient (κ) as appropriate. Fifteeen participants from each group were then selected for convenience according to their availability and completed the instrument a second time, following the same protocol applied during the first administration. The interval between the questionnaire administrations ranged from 7 to 14 days after the first session, with an average of 10 days (SD = 2.35). The QPAOT demonstrated excellent stability, with an ICC of 0.999 for both the NTA and AOT groups.

### Associations

No associations were found between QPAOT scores and age, sex, DMFT, or patients’ socioeconomic status in the NTA and AOT groups.

## Discussion

In recent years, research on instruments that assess quality of life has gained considerable interest because of the widespread emergence of instruments that differ in their development methods, content, application, and quality.^10^ Patients’ expectations regarding orthodontic treatment and their perceptions during treatment influence their satisfaction and success of treatment.^2,3,11^ The present study aimed to test the validity and reliability of the Brazilian version of the QPAOT, investigating the preservation of its psychometric properties for future research on the perceptions of Brazilian adolescents requiring orthodontic treatment and for use in clinical practice.

The methodology was designed to be similar to and comparable with that of the original questionnaire.^3^ As outlined in the original study, patients undergoing orthodontic treatment (AOT group) answered questions in the pain and functional impairment domains. During the translation and cross-cultural adaptation process, it was observed that patients who were about to start the treatment did not fully understand the targeted domains. Consequently, it was decided that only the motivation and treatment expectations domains would be administered to this group (NTA group).

Face validity refers to the extent to which a test or questionnaire appears to measure what it is intended to measure, based on a superficial inspection by experts or target respondents.^12^ It is a subjective judgment of whether the items seem appropriate and relevant for the construct being assessed and needs to be complemented by other constructs, such as content and construct validity, in addition to internal consistency. The questions from the validation domain were applied to the respective groups and their scores were used as a criterion for assessing face validity. That was considered an advantage of the present study because the time required to complete the questionnaire was reduced, and emphasis was given to the context experienced by the patients in each group.

An adjustment was made to the 0-10 visual scale in the questionnaire to ensure patients fully understood it and could provide reliable answers. As the patients did not show difficulties during the interviews and in the preliminary test, the questionnaire was applied in a self-administered manner during the pilot study, as suggested by the authors of the original English version.^3^ |Instrument stability was assessed using the test-retest strategy. Souza et al. stated that the test-retest strategy should be conducted between 10 and 14 days.^17^The interval between assessments should be short enough to prevent recall bias but not so long as to allow clinical changes to occur. In the present study, this interval was set between 7 and 14 days.

ICC values equal or greater than 0.7 were considered acceptable.^13^ Kappa values above 0.61 demonstrated a strong correlation and QPAOT showed stability across all items and domains. No association was found between the QPAOT and patient sex, corroborating the findings of Bos et al. and Prabakaran et al.^2,17^

No differences were found between female and male adolescents regarding orthodontic treatment, but girls showed a higher demand for treatment. The DMFT index did not influence patients’ perceptions of orthodontic treatment, likely due to the low DMFT indices found in the present sample. Patients’ socioeconomic status was not associated with motivation, expectation, pain, and functional impairment of adolescents before and during orthodontic treatment. These factors do not seem to interfere in quality of life, which corroborates the findings of Mandall and Palomares et al.^20,21^

Numerous questionnaires have been described in the literature to assess oral health-related quality of life (OHRQoL) of children and adolescents before, during, or after orthodontic treatment. Two systematic reviews with meta-analysis conducted by Javidi and Alrashed et al.^18,19^ found that there is a lack of standardized studies on the types of questionnaires used to assess quality of life in children and adolescents and highlighted the need for longitudinal studies, following patients before, during, and after treatment. The cross-sectional design and absence of a longitudinal follow-up precluded assessment of the predictive validity of the QPAOT. Similarly, the validity of the instrument was not compared with that of other established questionnaires, preventing the evaluation of concurrent validity. This could be a limitation of the present study, although the main purpose was to test whether the QPAOT could be applied to the Brazilian adolescent population.

Furthermore, due to sample size and design constraints, neither exploratory nor confirmatory factor analysis was performed, precluding a more in-depth investigation into the instrument’s construct validity. Future studies with larger and more diverse samples and longitudinal methodologies are essential to assess these important psychometric properties.

Despite these limitations, the present study contributes by offering a specific instrument designed to assess Brazilian adolescents’ perceptions of orthodontic treatment. The QPAOT can be applied to compare pain experiences across different orthodontic techniques and assist in understanding patient motivation, both of which are known to influence treatment success.^6,16,22^ In addition, the QPAOT provides insights into patients’ expectations, enabling orthodontists to foster stronger communication and improve patient cooperation.^1 1^ By measuring pain and functional limitations associated with various techniques, the instrument also supports clinicians in selecting the most appropriate for each case. In doing so, the instrument contributes to the delivery of patient-centered care and promotes higher quality outcomes, prioritizing the patient well-being.

## Conclusions

Based on the findings, the QPAOT demonstrated satisfactory face and construct validity, as well as internal consistency and stability, supporting its use as a reliable instrument to assess adolescents’ perceptions of orthodontic treatment. The findings indicate that adolescents’ perceptions before and during treatment were not significantly associated with age, sex, DMFT index, or socioeconomic status. A significant association, however, was observed with the DAI index: adolescents with a greater orthodontic treatment need reported higher motivation and expectations for treatment, along with increased levels of pain, discomfort, and functional limitations during the active phase. These findings underscore the importance of considering the degree of orthodontic treatment need when evaluating psychological and functional responses to treatment in adolescent populations.

## Data Availability

The authors declare that all data generated or analyzed during this study are included in this published article.
